# The effect of COVID‐19 stay‐at‐home order and campus closure on the prevalence of acute respiratory infection symptoms in college campus cohorts

**DOI:** 10.1111/irv.12837

**Published:** 2021-03-04

**Authors:** Oluwasanmi Adenaiye, Paul Jacob Bueno de Mesquita, Qiong Wu, Filbert Hong, Jianyu Lai, Shuo Chen, Donald K. Milton, Prometheus@UMD Consortium

**Affiliations:** ^1^ Maryland Institute for Applied Environmental Health University of Maryland College Park MD USA; ^2^ Department of Mathematics University of Maryland College Park MD USA; ^3^ Department of Epidemiology and Biostatistics University of Maryland College Park MD USA; ^4^ Division of Biostatistics and Bioinformatics School of Medicine University of Maryland Baltimore MD USA

**Keywords:** ARI, COVID‐19, influenza, lockdown, transmission mitigation

## Abstract

Evaluation of population‐based COVID‐19 control measures informs strategies to quell the current pandemic and reduce the impact of those yet to come. Effective COVID‐19 control measures may simultaneously reduce the incidence of other acute respiratory infections (ARIs) due to shared transmission modalities. To assess the impact of stay‐at‐home orders and other physical distancing measures on the prevalence of ARI‐related symptoms, we compared symptoms reported by prospective college cohorts enrolled during two consecutive academic years. ARI‐related symptoms declined following campus closure and implementation of stay‐at‐home orders, demonstrating the impact of population‐based physical distancing measures on control of a broad range of respiratory infections.

## 1 INTRODUCTION

As part of efforts to prevent the escalation of SARS‐CoV‐2 transmission, the US Centers for Disease Control and Prevention issued nationwide stay‐at‐home recommendations in the United States on March 15, 2020.^1^ Also, states across the country issued varying degrees of physical distancing measures, including restrictions on transportation and movement, school closures, and business closures. Since then, studies have shown, using mathematical and epidemiological models, that such distancing measures likely reduced transmission of COVID‐19.^2^, ^3^ Others have shown the effect of these measures on the incidence of other viral acute respiratory infections (ARIs) such as influenza.^4^, ^5^ However, only a few community surveillance studies were ongoing before the stay‐at‐home orders and can show the effect of these measures on a population already under ARI surveillance. Here, we use data from a prospective college cohort under ARI surveillance to assess the impact of the physical distancing measures on the rate of ARI‐related symptoms.

## 2 METHODS

**FIGURE 1:**
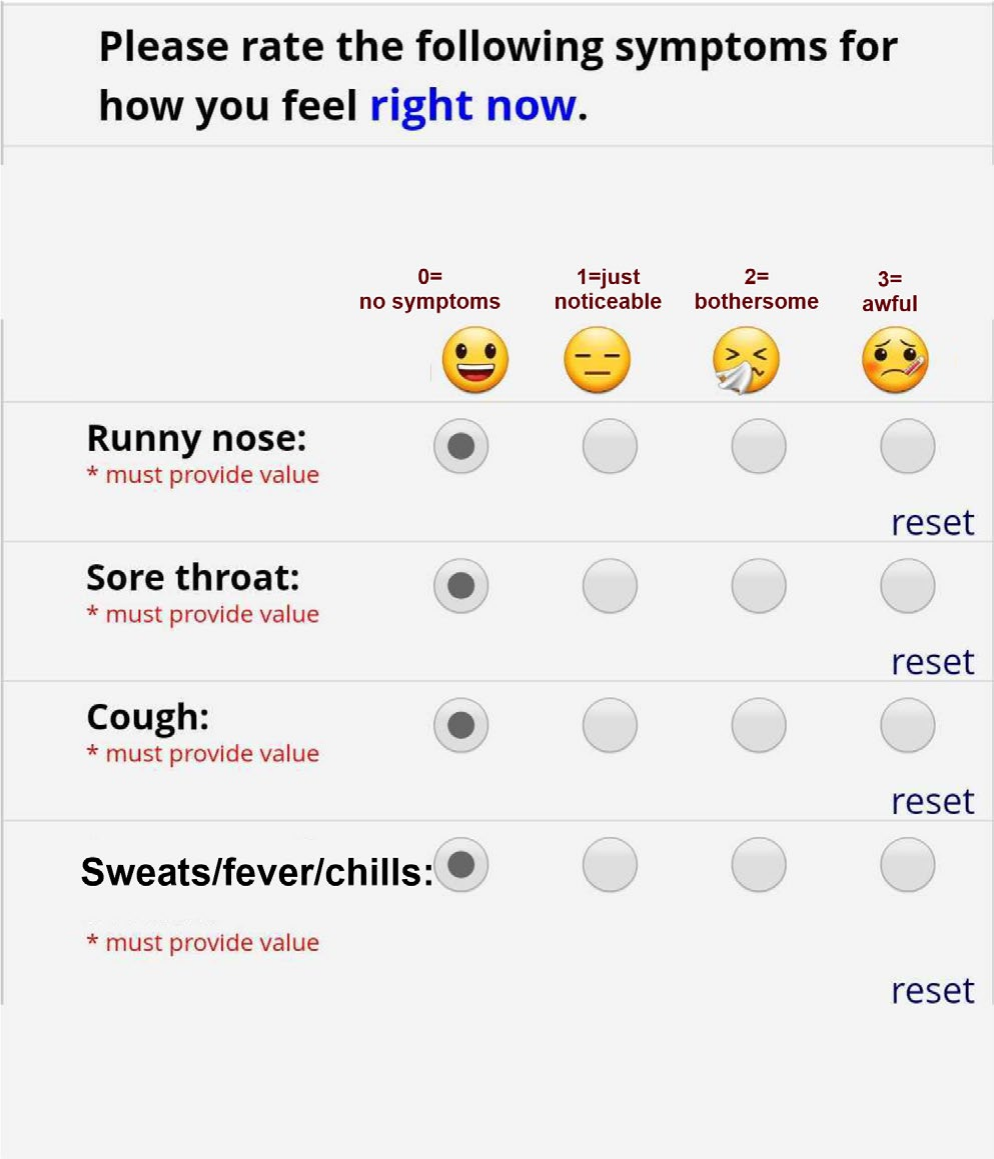
The daily symptom survey that was sent to study participants. The symptom score is rated as follows, 0 – No symptoms, 1 – Just noticeable, 2‐ Bothersome, 3 – Awful

We recruited new cohorts of college students and staff each academic year for 4 years and monitored them prospectively for the occurrence of ARI (2017‐2020). At the beginning of the study, we asked participants to complete an online survey on their demography and health history and to provide baseline biological specimens. Participants were then prospectively monitored for ARI. In the most recent 2 years (2019 and 2020), we monitored the cohorts for ARI‐related illness using symptom surveys sent daily at 10 am via text message to all enrolled participants. Participants were asked to rate each of the ARI‐related symptoms on the daily text survey on a severity scale of 0‐3 (3 = most severe), based on how they felt at the time of their response (Figure 1). The daily symptom surveys were part of a larger study wherein participants with ARI‐related symptoms were invited to the study clinic to provide mid‐turbinate (MT) nasal swab specimens. Swabs were tested for 44 respiratory pathogens using a TaqMan Array Card® (Thermo Fisher, Waltham, MA, USA).^6^ Physical environmental conditions in the living spaces of the participants who tested positive and their contacts were monitored as described by Zhu et al^7^

The data presented in this report are from the daily symptom surveys for two consecutive spring semesters, 2019, and 2020, when participants were prompted daily to report ARI symptoms. During the 2019 spring semester, we performed a daily and a weekly lottery among all respondents, and a randomly selected participant from each lottery received $20 or $100, respectively. To test the effect of payment on survey completion, we performed a counterfactual analysis, matching each lottery winner with an unrewarded control participant with the same number of responses during the week prior to winning, and compared their response rates in the following week (SI Methods). We discovered that the rewarded participants, versus the unrewarded participants, had about a 20% higher response rate to symptom surveys and over 3 times the odds of responding in the days following reward. For this reason, we updated the compensation framework for the 2020 spring semester so each participant was compensated $1 per day for completing the symptom survey, plus $5 on a random day in a month if they completed the survey on that day.

We created two indicator variables for ARI‐related symptoms for each person‐day observation: (a) sum of symptom scores >3 and (b) presence of self‐reported fever, with cough or sore throat. A 3‐day simple‐moving‐average (SMA) was constructed with SMA package^8^ from the percentages of respondents who met the criteria for each of the two categories. Plots were created with ggplot2^9^ to compare the trends before and after stay‐at‐home orders. To account for differential average response rates, we calculated the proportion of enrollees who reported symptoms in each of the two symptom categories. Categorical variables were compared across cohort years with chi‐squared tests and continuous variables were compared with Mann‐Whitney *U* tests. We tested, using one‐dimensional scan statistics,^10^ to evaluate differences between the reporting rates of the two groups of ARI‐related symptoms between the years 2019 and 2020 (SI).

## 3 RESULT

**FIGURE 2:**
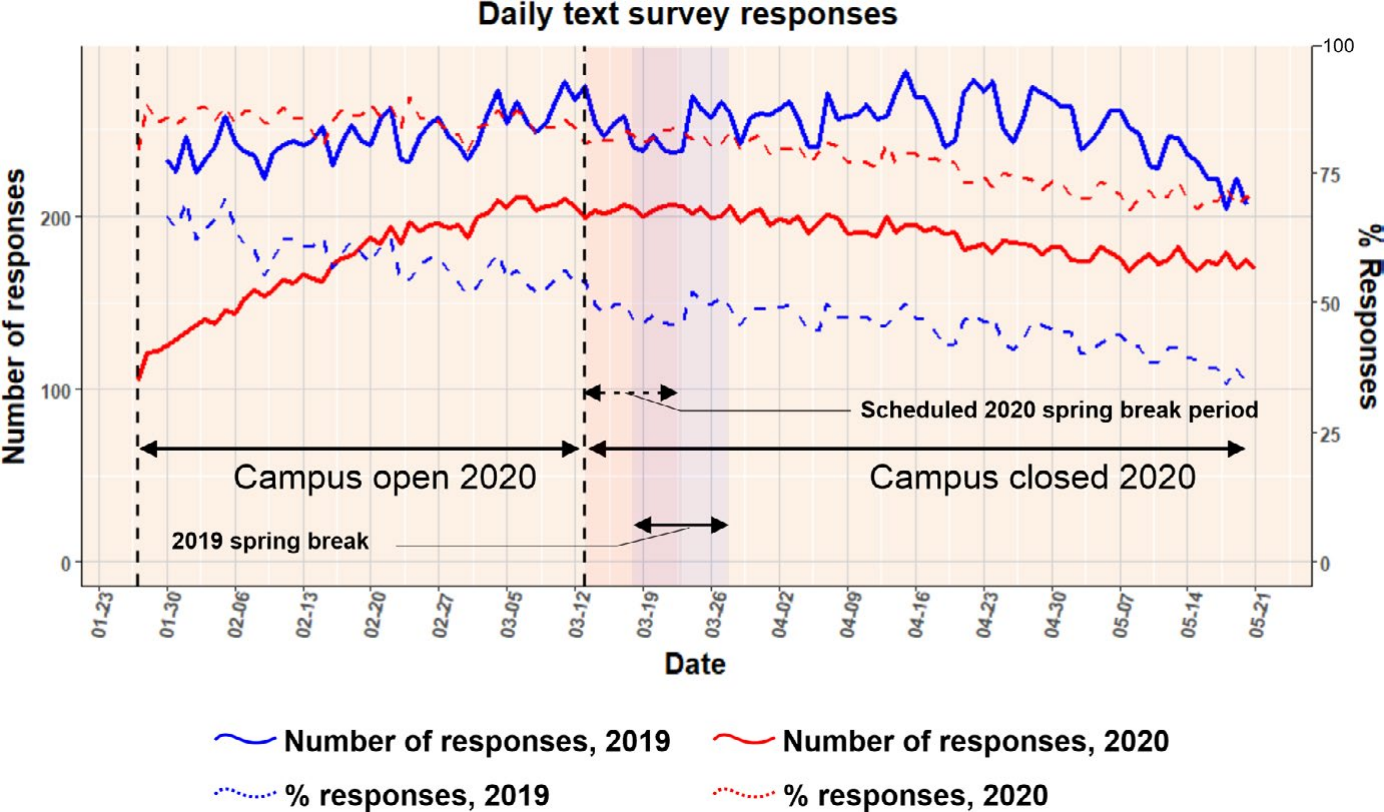
The daily trend of the number of responses received and the proportion of the enrollees that responded to the daily text. As more people enrolled in the study, the number of sent surveys increased. The campus closed for spring break on March 13, 2020, and remained closed due to the COVID‐19 pandemic, till the end of the surveillance period on May 21, 2020

**FIGURE 3:**
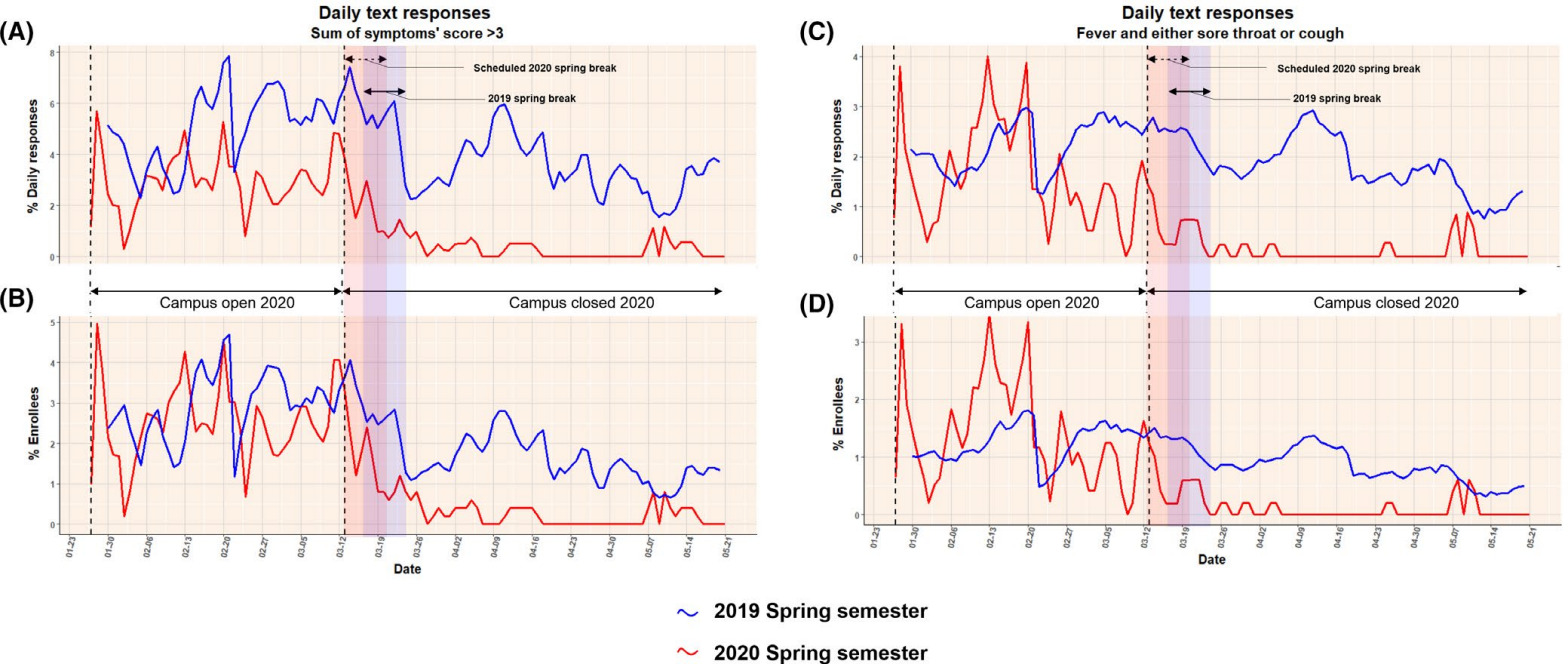
The daily trend of the proportions of respondents and enrollees with ARI‐related symptoms for the two academic semesters. A, The proportion of daily respondents reporting a sum of symptoms’ score >3. B, The proportion of the enrollees reporting a sum of symptoms’ score >3. C, The proportion of respondents reporting having a fever and either a sore throat or cough, D. The proportion of the enrollees that reported having a fever and either a sore throat or cough. The campus closed on March 13, 2020, and did not reopen during the study due to COVID‐19

For the spring semester of 2019, daily symptom surveillance commenced on January 30, 2019, and ended on May 21, 2019. For the spring semester of 2020, the daily symptom surveillance for some cohort members started in December, but the majority enrolled in January and the data in this report includes reports starting on January 27 and ending on May 21, 2020. The characteristics of respondents are summarized in Table 1. Seventy‐four percent of the participants who were monitored in 2020 lived on‐campus during the period when the campus was open and residence halls were occupied. The majority recorded Maryland as their state of permanent residence. Figure 2 shows the daily trend in the number of participants and the proportion of enrollees that responded to the daily text. Figure 3 shows the daily trend of the proportions of respondents and enrollees with ARI‐related symptoms for the two academic semesters. Figures S1 and S2 show the epidemic curve of the infections that were detected weekly in the participants who were tested in the 2019 and 2020 cohorts respectively.

**TABLE 1:** Characteristics of respondents

	2020	2019
Surveillance period	January 27–May 21	January 30–May 21
Number of participants sent daily survey^a^		
Mean ± SD (Range)	230.2 ± 31.2 (132‐249)	507.6 ± 78.4 (349‐596)
Number of daily respondents		
Mean ± SD (Range)	182.7 ± 22.1 (106‐211)	250.1 ± 15.7 (204‐284)
Survey completion rate ± SD^b^	79.8% ± 5.9	50.4% ± 8.1
Age in years		
Mean ± SD	20.4 ± 3.3	19.9 ± 2.7
Female (%)	150 (60.2%)	339 (56.9%)
On‐campus residents (%)	183 (73.5%)	476 (79.8%)
Residents of the State of Maryland (%)	225 (90.4%)	372 (82.4%)

Note

During the 2020 spring semester, each participant was compensated $1 per day, plus $5 on a random day in a month, for completing the daily symptom survey, whereas during the 2019 spring semester, we performed a daily and a weekly lottery among all respondents, and the randomly selected participant from each lottery received $20 and $100, respectively.

Abbreviations: SD, Standard Deviation.

^a^ This number represents the number of participants that were receiving daily text survey messages. It increased as more people enrolled in the study.

^b^ Survey completion rate, 2020 vs 2019; *P*‐value (<.0001).

Within the 2019 cohort, matching on response rate in the prior week, participants who received a lottery payment had a 3.13‐fold (*P* < .001) greater response rate in the following week than those who did not receive payment. The average completion rate of the daily symptom survey was 80% in the 2020 semester and 50% in 2019 (*P* < .0001). There were no observed significant differences in age (*P* > .79) and sex (*P* > .37) distributions between the participants in the 2019 and 2020 cohorts. No significant differences were observed between the respondents and non‐respondents in 2019 (Age: *P* > .89, sex: *P* > .67).

The ARI‐related symptoms detection rate was reduced over the course of the semester in each of the two years. The drop in ARI‐related symptoms fell at a higher rate in 2020 compared to 2019, and the drop to minimal ARI‐related symptoms detection rate persisted in 2020. There was a steady decrease in the rate of ARI‐related symptoms starting from around March 16, 2020. When compared to 2019, we observed a significant reduction in the reporting rates of symptoms with a sum of scores >3 (*P* < .05) and reporting rates of fever with cough or sore throat (*P* < .0001) starting from March 31 and April 1, respectively (Figures S3 and S4). There was no significant difference in the reporting rates of the ARI‐related symptoms between the two years before campus closure for either set of symptoms (*P* ≥ .46).

## 4 DISCUSSION

We showed variations in the prevalence of ARI‐related symptoms in two college campus cohorts monitored for ARI in the spring semesters of two consecutive academic years. The members of the 2020 cohort with a low prevalence of ARI‐related symptoms spent a considerable period observing stay‐at‐home orders with presumably reduced physical contacts. The members of the 2019 cohort spent the surveillance period living in a large university campus with high numbers of daily interactions and a much higher occupant density setting.

The period of a significant decrease in the prevalence of ARI‐related symptoms in the Spring‐2020 cohort started from around March 31, 2020; eighteen days after the campus was closed on March 13 and when physical distancing measures were introduced in the state of Maryland where most of the participants live.^11^, ^12^ The difference between the two semesters was no longer significant after April 18 in part due to falling symptom rates in late spring during the reference period (2019) which we believe is because of the decreasing prevalence of influenza infections (Figures S1 and S2). The general decrease in 2019 in ARI detection over semester could be related to the seasonality of many ARIs with higher incidence generally observed in the winter compared with the summer season in temperate climates. We did not observe a substantial reduction in the number of daily survey completions following campus closure in the year 2020.

The 3.13‐fold increase in odds of responding in the week following lottery payments in 2019 winners, compared with unrewarded controls, suggested that payment was an effective reinforcement strategy to promote survey‐completing behavior. We believe this resulted in the higher response rate in 2020 when every participant was rewarded for daily survey completion, compared to 2019 when we made payments only to participants who won the lottery. Increasing the response rate in 2020 based on the lessons learned during the 2019 cohort was the goal of modifying the compensation framework.

Because we adjusted for the response rate in the analysis comparing ARI‐related symptom detection between cohort years 2019 and 2020, we do not believe that these differences in response rate affected the main analysis.

This report offers evidence for the effectiveness of physical distancing measures and campus closure on reducing the incidence of ARI in the campus community. This may explain the large reduction in the number of influenza cases observed in the southern hemisphere in 2020 compared to the same calendar period in 2019.^13^ Furthermore, our findings demonstrate the impact of population‐based physical distancing measures on control of a broad range of respiratory infections.

## CONFLICT OF INTEREST

The authors of this paper have no conflict of interest to declare.

## AUTHOR CONTRIBUTIONS

**Oluwasanmi Adenaiye:** Conceptualization (equal); Data curation (lead); Formal analysis (lead); Methodology (equal); Visualization (lead); Writing – original draft (lead); Writing – review and editing (equal). **Paul Jacob Bueno de Mesquita:** Conceptualization (equal); Methodology (equal); Writing – original draft (equal); Writing – review and editing (equal). **Qiong Wu:** Data curation (equal); Formal analysis (equal); Methodology (equal); Visualization (equal); Writing – original draft (supporting). **Filbert Hong:** Conceptualization (equal); Data curation (lead); Methodology (equal); Project administration (lead). **Jianyu Lai:** Conceptualization (equal); Writing – original draft (supporting); Writing – review and editing (supporting). **Shuo Chen:** Conceptualization (equal); Formal analysis (equal); Methodology (equal); Supervision (equal); Writing – review and editing (equal). **Donald K. Milton:** Conceptualization (lead); Funding acquisition (lead); Methodology (lead); Supervision (lead); Writing – review and editing (lead).

### PEER REVIEW

The peer review history for this article is available at https://publons.com/publon/10.1111/irv.12837.

## Supporting information

Supplementary Material

## Data Availability

The data that support the findings of this study are available on request from the corresponding author. The data are not publicly available due to privacy or ethical restrictions.
