# Supplementary material for: The effect of COVID‐19 stay‐at‐home order and campus closure on the prevalence of acute respiratory infection symptoms in college campus cohorts
Source: Influenza Other Respir Viruses. 2021 Mar 4;15(3):331–5. doi: 10.1111/irv.12837 (PMC8014755; doi:10.1111/irv.12837)
Supplement: Supplementary file 1 — Supplementary Material [file IRV-15-331-s001.docx]

Supplemental Information (SI) for “The effect of COVID-19 stay-at-home order and campus closure on the prevalence of acute respiratory infection symptoms in college campus cohorts.”

Oluwasanmi Adenaiye^1^, P. Jacob Bueno de Mesquita^1^, Qiong Wu^2^, Filbert Hong^1^, Jianyu Lai^1,2^, Shuo Chen^3^, Donald K. Milton^1*^ and Prometheus@UMD Consortium

^1^Maryland Institute for Applied Environmental Health

_­_^2^Department of Epidemiology and Biostatistics

University of Maryland, College Park, MD 20742, USA

^3^Division of Biostatistics and Bioinformatics, School of Medicine, University of Maryland, Baltimore, MD, USA

Contents

[Prometheus Consortium Team Members 1](#_Toc51005004)

[Supplemental Methods 1](#_Toc51005005)

[Supplemental Results 2](#_Toc51005006)

[Supplemental Figures (S1-S4) 3](#_Toc51005007)

### Prometheus Consortium Team Members

### Donald Milton, Jelena Srebric, Ashok Agrawala, Filbert Hong, Tianzhou (Charles) Ma, Sheldon Tai, Somayeh Youseffi, Barbara Albert, Jennifer German, Rhonda Washington-Lewis, Yi Esparza, Faith Toure, Delwin Suraj, Christian Calilung, Joann Prosser, Mara Cai, Prathima Devanath, Shengwei Zhu, Nicholas Matisse, Derek Yarnell, Edward J. Ridge, Shawn Bobbin, P. Jacob Bueno de Mesquita, Oluwasanmi Adenaiye, Jianyu Lai, Dewansh Rastogi, Qiong Wu, Kofi Addo, Jinay Shah, Sebastian Romo, Heba Aly, Faizan Wajid, Liam Monahan, Mathew Frieman, Emmanuel Mongodin, Shuo Chen, Wilbur Chen, Phil Felgner, Saahir Khan, Al Jasinskas, Rie Nakajima, Aarti Jain, Dan Nasko, Todd Treangen, Leo Elworth, Adam Porter, Sandro Fouche, Madeline Diep, Sigurthor Njorgvinsson, Duncan Woodbury, Richard Martinello.

### Supplemental Methods

We tested the effect of winning the lottery once on daily survey completion behavior. To control for the effect that participants who have won the lottery generally respond more, we matched each winner with a control participant (who never won the lottery) by previous behavior. For each instance of lottery winning, the response rate during the 7 days before the win date was calculated, and a control subject with the same response rate during the same 7-day period was identified as a match (e.g., if the win date is 2019-04-26, the response rate during 2019-04-20 to 2019-04-26 would be considered). Wins occurring within 7 days of enrollment were excluded from the analysis. The responses during the 5 days after the win date were considered as binomial, and a generalized estimating equation was used to take into account repeated measures and to isolate the influence of lottery reward on subsequent, short-term (5 days) survey response likelihood (e.g., if the win date is 2019-04-26, Day 1 is 2019-04-27, and Day 2 is 2019-04-28, etc.). If the win occurred within 5 days before dropping out of the study, it was excluded from the analysis. If the time interval between two wins is less than or equal to 5 days, these two wins will be considered as one (e.g., if the first win date is 2019-04-26 and the second is 2019-04-28, the 7 days between 2019-04-20 and 2019-04-26 would be used for matching, and 5 days between 2019-04-29 and 2019-05-03 would be compared between the winner participant and matched control participant.)

We also tested the effect of the “stay-at-home” policy after the spring-break in 2020. The stay-at-home policy went into effect a day before spring break, thus overlapping with the spring break. Therefore, to separate the temporal effect confounding the effect of the stay-at-home (e.g., whether there is a natural difference before and after spring break within a calendar year), we capitalize on the self-reported data of 2019 and 2020. Specifically, we test the contrast that [2019_post spring break – 2020_post spring break] - [2019_pre spring break – 2020_pre spring break] (Figure S3, S4).

Under the null hypothesis [H_0_] that the “stay-at-home” policy has no effect on symptom reporting rate, the difference of symptom reporting rates between 2019 and 2020 would remain at the same level before and after the spring break. However, if we observe that i.) the symptom-report-rates show no difference before the spring break in 2019 and 2020 and ii.) there is a temporal period after spring break when the symptom reporting rates in 2020 are systematically and significantly lower than 2019 [H_1_], then we reject the null and conclude that the “stay-at-home” policy reduced the self-reported symptom rate.

We test the hypothesis by applying the one-dimensional temporal scan statistics and observed the p-values using permutation tests.^1^ We control the family-wise error rate at the level of 5%.

### Supplemental Results

#### The results of the counterfactual analysis show the estimated 5-day post lottery winning response rate for the lottery winning participants is 0.866 compared with 0.674 for matched, unrewarded control participants (p<0.0001). Compared with the controls, lottery winners had 3.13 times the odds of responding during the 5-day post-lottery winning period.

#### When compared with Spring 2019, the longest period during which there was sustained reduced reporting rates of symptoms with sum of scores greater than 3 was 12 days starting from March 31 (Figure S1) and 18 days starting from April 1 for symptoms of fever with cough or sore throat (p<0.05, p<0.0001 respectively). The time interval during which there was sustained reduced symptom reporting rates before the campus closure lasted 5 days for symptoms with sum of scores >3 and was 7 days for symptoms of fever with cough or sore throat but both were not significant following permutation tests (p≥0.46).

**SI References**

1. Gotway CA, Waller LA. *Applied Spatial Statistics for Public Health Data*. Vol 368. John Wiley & Sons; 2004.

### Supplemental Figures (S1-S4)

###
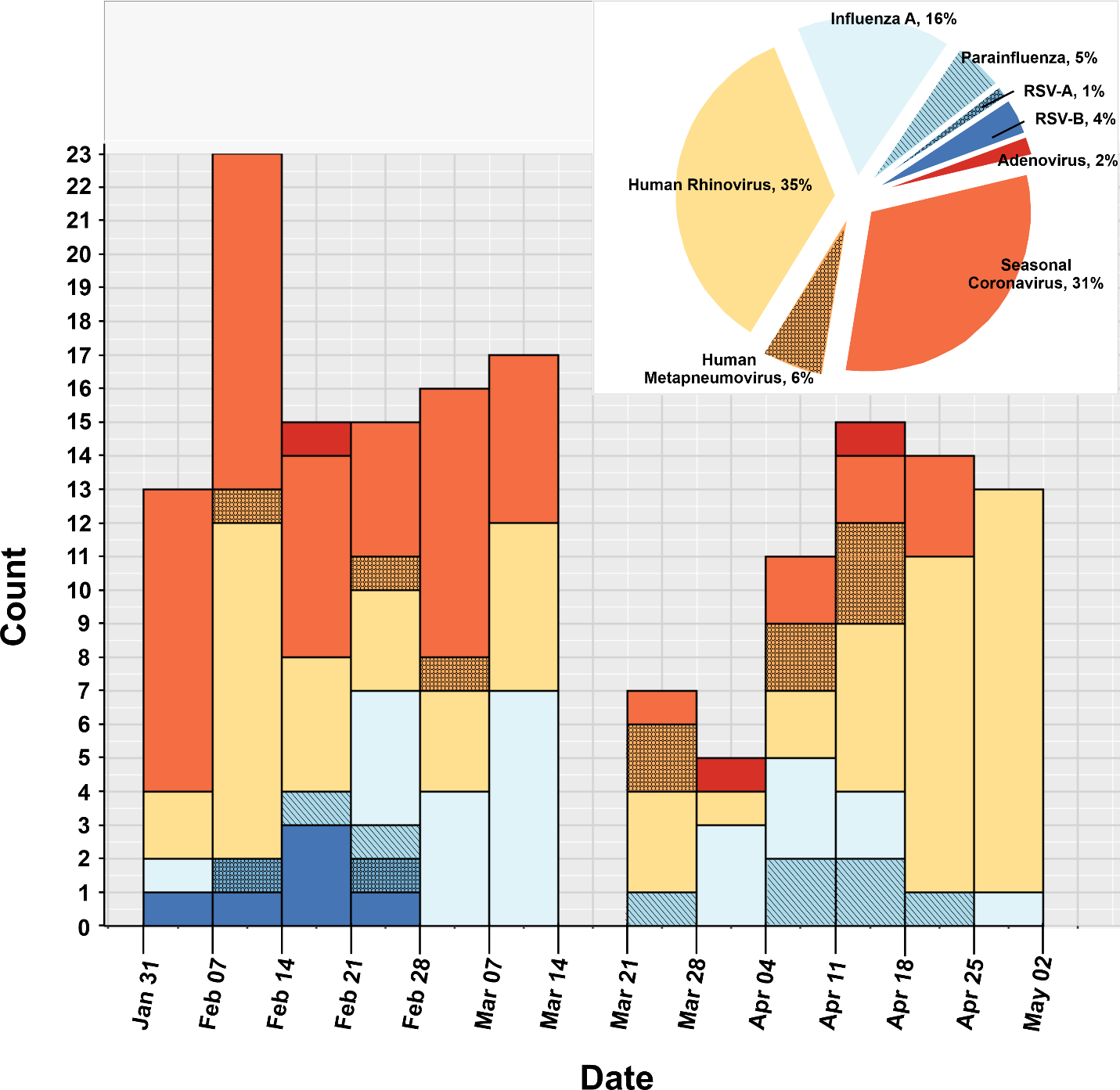


**Figure S1.**

Plot showing the viral infections that were detected in the 2019 study cohort. Seasonal coronavirus includes the strains of Coronavirus (OC43, 229E, HKU1 and NL63) that are known to cause common cold.


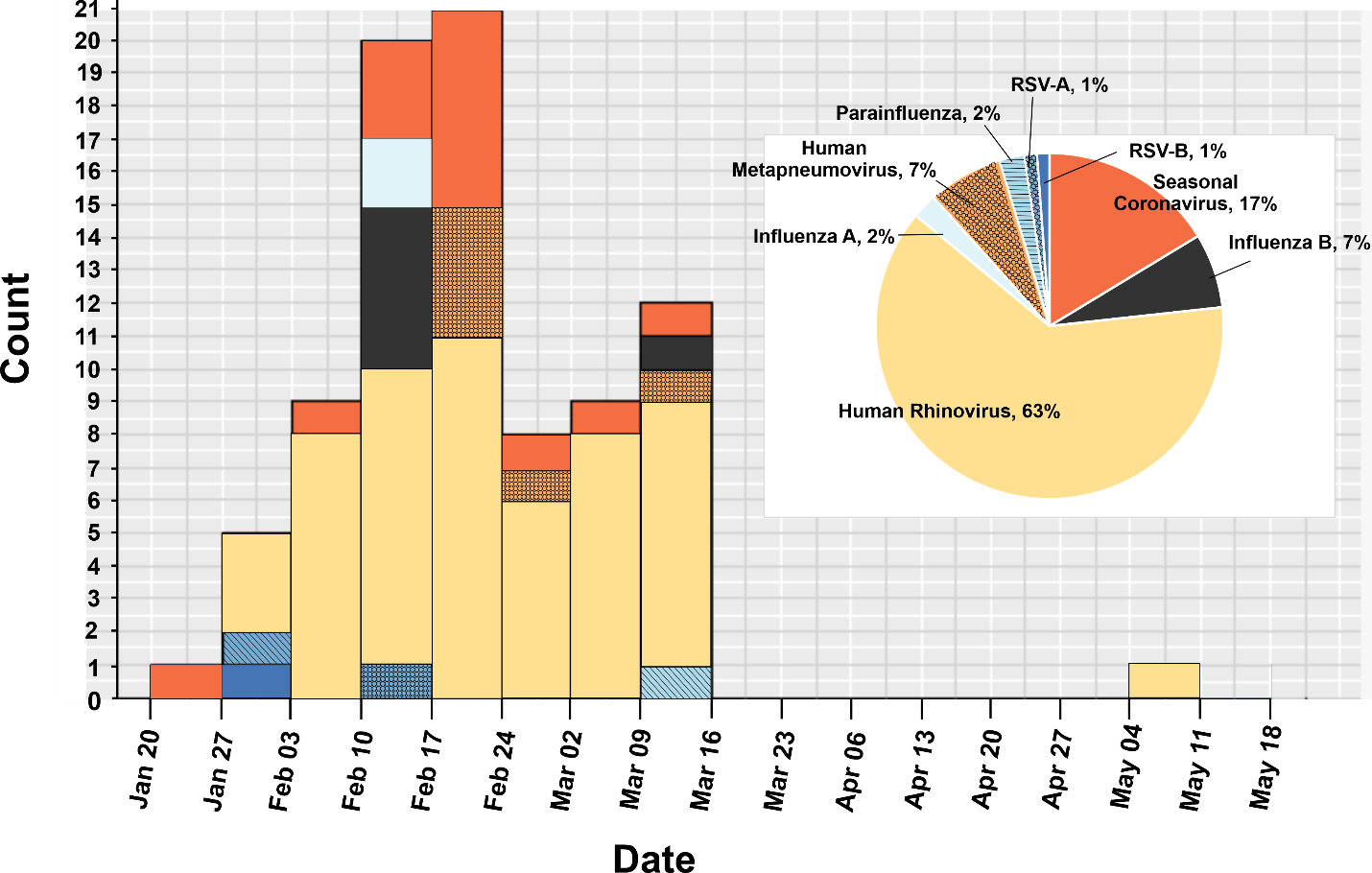


**Figure S2.**

Plot showing the viral infections that were detected in the 2020 study cohort. Due to campus closure on March 13, no in-person study visit was conducted until May 6. Seasonal coronavirus includes the strains of Coronavirus (OC43, 229E, HKU1 and NL63) that are known to cause common cold.


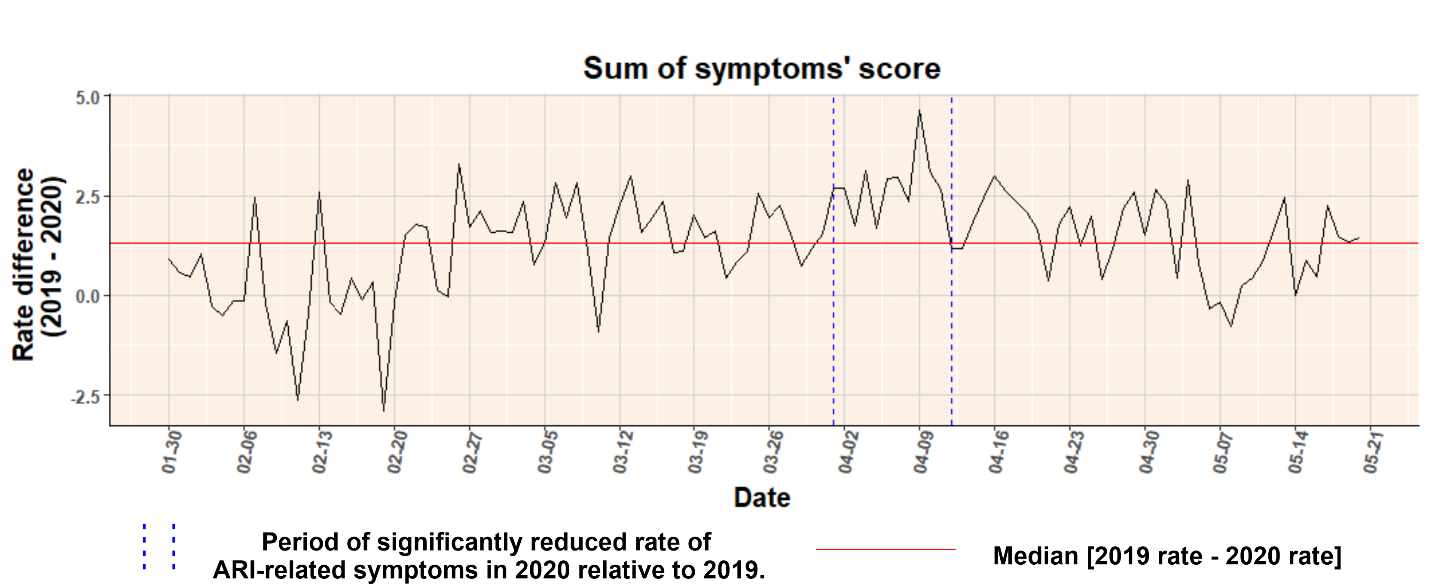


**Figure S3.** Plot showing the difference in the rate of reporting symptoms of total scores > 3 in Spring-2019 minus Spring-2020. The blue markings show the period when the reported rates were significantly reduced in the Spring-2020 cohort compared to Spring-2019 cohort and occurred starting from March 31 and lasted through April 11. The red line indicates the median of the difference in the rate of reported symptoms.


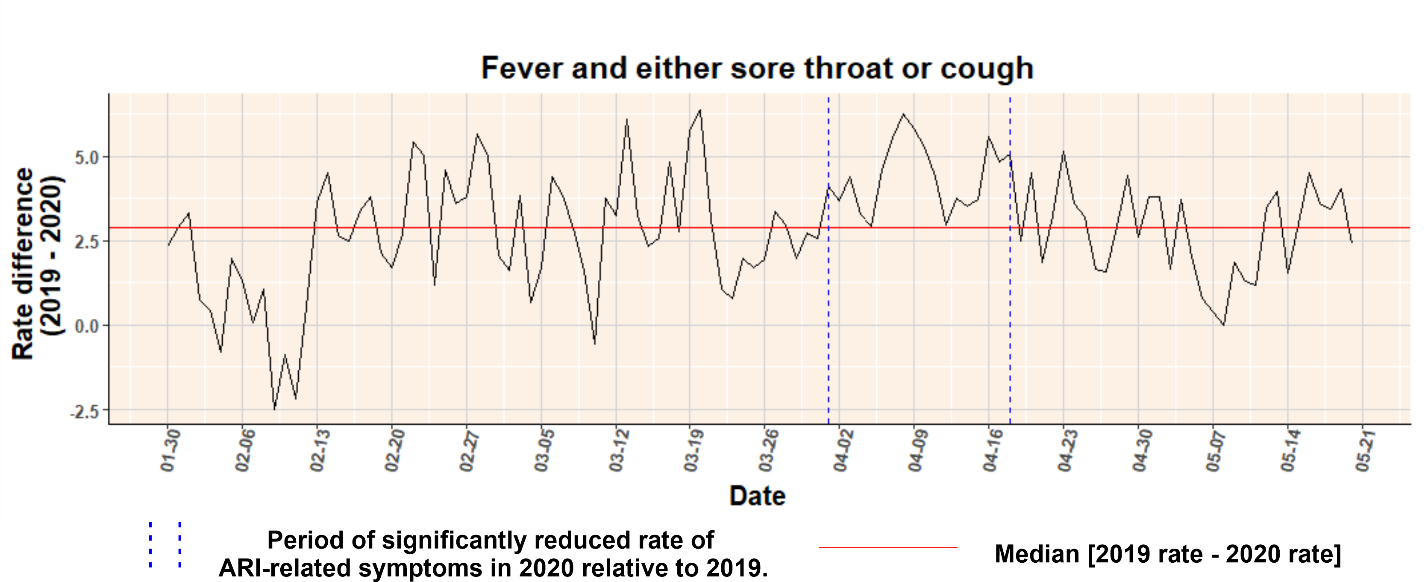


**Figure S4.** Plot showing the difference in the reporting rates of fever with cough or sore throat in Spring-2019 minus the rate in Spring-2020. The blue markings show the period when the reported rates were significantly reduced in the Spring-2020 cohort compared to Spring-2019 cohort and occurred starting from April 1 and lasted through April 18. The red line indicates the median of the difference in the rate of reported symptoms.
